# Genomic insights into a clade-specific *Candida tropicalis* lineage with resistance to azoles and immune evasion traits

**DOI:** 10.1128/mbio.00235-26

**Published:** 2026-03-11

**Authors:** Kusum Jain, Nityendra Shukla, Amtoj Kaur, Barsha Kalita, Shriya Srivastava, Suhani Yadav, Preeti Rani, Poornima Kagra, Ashutosh Singh, Komal Chaudhary, Hardeep Kaur, Jenish Patel, Manju Chauhan, Bansidhar Tarai, Jitendra Narayan, Neeraj Chauhan, Anuradha Chowdhary

**Affiliations:** 1 Medical Mycology Unit, Department of Microbiology, Vallabhbhai Patel Chest Institute, University of Delhi 28742, Delhi, India; 2 Department of Microbiology and Molecular Biology, Max Super Speciality Hospital 76177, New Delhi, India; 3 Department of Zoology, Ramjas College, University of Delhi 28742, Delhi, India; 4 Center for Discovery and Innovation, Hackensack Meridian Health 3139, Nutley, New Jersey, USA; 5 CSIR- Institute of Genomics and Integrative Biology, New Delhi, India; 6 National Reference Laboratory for Antimicrobial Resistance in Fungal Pathogens, Vallabhbhai Patel Chest Institute, University of Delhi 28742, Delhi, India; Instituto Carlos Chagas, Curitiba, Brazil

**Keywords:** *ERG11*, clade 4, India, MLST, ALS, whole-genome sequencing, metabolomic, biofilm, RNA-Seq

## Abstract

*Candida tropicalis* is a leading cause of invasive candidiasis in the Asia-Pacific region with reported crude mortality rates exceeding 50%. The rising prevalence of azole-resistant strains presents a significant clinical challenge. We analyzed 1,016 *C. tropicalis* clinical isolates collected over nine years from 27 hospitals across North India. Fluconazole resistance was detected in 5.1% (*n* = 52) of isolates, with cross-resistance observed to voriconazole in 55.7% and itraconazole in 44.2% of isolates. Multilocus sequence typing (MLST) analysis of global 1,630 isolates including 208 Indian and whole-genome sequencing of 716 global isolates (139 Indian) confirmed the clonal emergence and persistence of azole-resistant MLST clade 4 strains in Indian hospitals. Phylogenomic analyses identified that Indian azole-resistant lineage was closely related to azole-resistant isolates from mainland China and Taiwan. The underlying mechanism of resistance involved hotspot mutations (Y132F) in the *ERG11* gene along with its duplication, overexpression, and twofold high ergosterol content. Comparative transcriptomics of two clinical isolates exhibiting >512 fold difference in fluconazole susceptibility identified upregulation of virulence-associated genes, *ALS7* gene (eightfold), *SAP7* and *SAP9* (1.6- and 2-fold, respectively) in azole-resistant isolate. Furthermore, azole-resistant isolates showed robust biofilm-associated metabolic activity (twofold), reduced β-glucan exposure, and greater survival in both neutrophil and macrophage killing assays. Notably, azole-resistant lineage exhibits several traits associated with adhesion and immune evasion that could possibly enable its spread in healthcare settings and signals the beginning of a greater spread of this clone. The urgent need for continuous genomic surveillance and antifungal stewardship is warranted to mitigate the spread of multidrug-resistant *C. tropicalis*.

**IMPORTANCE:**

Invasive fungal infections affect 6.5 million people annually and are associated with high mortality rates. Despite being the leading cause of invasive yeast infections in the Asia-Pacific, this is the first comprehensive study of *Candida tropicalis* from India documenting the emergence of azole-resistant clonal lineage (clade 4) in several hospitals in India. Azole resistance is driven by mutations, gene duplication, and overexpression of its target gene *ERG11*. The Indian azole-resistant isolates showed high genetic relatedness with those from China. Also, resistant isolate showed overexpression of virulence-related genes and robust biofilm formation. Notably, reduced β-glucan exposure in fluconazole-resistant isolates may contribute to their decreased susceptibility to the innate immune system. Importantly, this study provides evidence for the emergence of azole-resistant *C. tropicalis* lineage in India, which exhibits several traits associated with adhesion and immune evasion that could possibly enable its spread in healthcare settings leading to a public health concern.

## INTRODUCTION

*Candida* species cause a broad spectrum of diseases, ranging from superficial to life-threatening invasive candidiasis (IC) (1). Candidemia is the most frequent presentation of IC, accounting for up to 10% of hospital-acquired blood stream infections (BSIs) (2). *Candida* infections are associated with prolonged hospitalizations, high healthcare costs, and increased morbidity and mortality (3, 4). The spectrum and prevalence of *Candida* species causing invasive diseases varies in different geographical regions. *Candida albicans* has long been the most prevalent species (5). However, a global trend toward an increase in non-*albicans Candida* (NAC) species with a corresponding increase in drug resistance leading to treatment challenges, including increased costs and prolonged hospital stays, has been a matter of concern in *Candida* infections (6). Among NAC infections, multidrug-resistant (MDR) species, including *Candida parapsilosis*, *Nakaseomyces glabratus* (formerly *Candida glabrata*), and *Candida auris*, have been recognized as global health threats (6). However, in large regions of the world, that is, Southeast Asia and Latin America, *Candida tropicalis* remains the first or the second most important cause of *Candida* BSIs, after *C. albicans*, highlighting the need for vigilant surveillance (5, 7–12). *Candida tropicalis* is ubiquitous in the environment and widely isolated from agriculture fields, forest soil, sea water, and rivers and from surfaces of fruits from China, Taiwan, Brazil, and India (13). Furthermore, this yeast can tolerate high salt concentrations, high temperatures, and oxidative stress and displays efficient adhesion and biofilm-associated growth, which facilitates its environmental survival, especially in tropical regions (14, 15). Importantly, in the last two decades, azole resistance in *C. tropicalis* has increased worldwide (5, 16, 17). Azole antifungals are commonly used for prophylaxis in patients with neutropenia and are the mainstay of treatment for candidemia in low- and middle-income countries where echinocandins’ availability is limited. Particularly, access to fluconazole (FLU) across Africa and the Asia-Pacific region is >90%, while access to micafungin (MFG) is limited in African hospitals (22.5%) and in the Asia-Pacific region (56%) (18). The emergence of azole resistance in *C. tropicalis* raises concern for treatment in settings with limited resources (19).

The SENTRY Program analyzed 20,788 invasive isolates of *Candida* (37 species) collected from 135 medical centers in 39 countries spanning two decades (1997–2016). The highest rates of fluconazole-resistant (FLU-R) isolates were seen in *N. glabratus* from North America (10.6%) and *C. tropicalis* from the Asia-Pacific region (9.2%) (5). Furthermore, in *C. tropicalis*, resistance to FLU increased from 2.5% to 4.9% from 1997 through 2014 (5). Also, reports from Australia showed an increase in FLU-R from <2% to 16.7% over a decade in cases of candidemia (16, 17). Similarly, data from the China Invasive Fungal Resistance Monitoring Network showed an increasing FLU-resistance rate of *C. tropicalis* from 11.2% in 2009 to 42.7% in 2014 (20). Recently, a high rate of FLU-R in *C. tropicalis* has been reported from Algeria (31.5%), Belgium (20%), Canada (12%), and Denmark (6%) in cases of candidemia (21–27). Particularly in Asia, predominately the data from China indicate that the emergence of azole resistance in *C. tropicalis* is linked to the expansion of a specific genetic lineage (multilocus sequence typing [MLST] clade 4) carrying A395T/W mutations in the azole target gene *ERG11* conferring a key substitution Y132F (9). However, outbreaks involving patient-to-patient transmission of MLST clade 4 in the hospital settings are lacking.

In India, *C. tropicalis* accounts for 41.6% cases of candidemia and is the leading cause of candidemia in adult intensive care units (ICUs) (7, 8, 12). Considering the high burden of *C. tropicalis* infections in the Indian subcontinent, there is a significant gap in comprehensive studies exploring the genetic population structure and mechanisms of azole resistance across a substantial clinical data set. Thus, the population dynamics and evolutionary trajectories of *C. tropicalis* in this region remain poorly understood. This study uses whole-genome sequencing (WGS) and multilocus sequence typing (MLST) to analyze the genetic diversity and population structure of clinical and environmental *C. tropicalis* isolates collected from 2014 to 2022. Additionally, through transcriptomic profiling, gene expression, and ergosterol quantification assays, this study investigates key molecular mechanisms driving azole resistance. We found that azole-resistant (AZO-R) *C. tropicalis* clone (MLST clade 4) is emerging in the hospital settings. Moreover, these resistant strains exhibit increased biofilm formation and enhanced resistance to neutrophil-mediated killing, underscoring the urgent need for effective intervention strategies.

## RESULTS

### *In vitro* antifungal susceptibility profile and predominance of Y132F substitution in the azole target Erg11 of fluconazole-resistant *Candida tropicalis* isolates

A total of 1,016 clinical isolates of *C. tropicalis* were collected over a 9-year period (2014–2022) from 27 hospitals across Delhi and the adjoining areas of the National Capital region (NCR), including adjacent districts of three states of India (Uttar Pradesh, Haryana, and Rajasthan), as a part of an ongoing antifungal surveillance program of all yeast isolates cultured from clinical samples. In our antifungal surveillance (2014–2022), *C. tropicalis* ranks first, accounting for 34% (*n* = 1,016/2,998) isolates, followed by *C. auris* (23%). Additionally, 16 environmental isolates from the surface of fruits and the inanimate hospital environment were included in the study. Two-thirds of the clinical isolates (64.2%; *n* = 652) were obtained from BSIs, followed by isolates from urine (21.7%, *n* = 221), respiratory tract (10%; *n* = 102), and vaginal swabs (1.7%; *n* = 18). The remaining 2.4% (*n* = 23) of isolates were obtained from body fluids and biopsy samples. Of these candidemia isolates, 39.7% were recovered from the medical ICU, followed by the pediatric ICU (37.9%), neonatal ICU (12%), and surgical ICU (10.4%). The minimum inhibitory concentration (MIC) data of 1,016 *C. tropicalis* isolates showed 5.1% (*n* = 52/1,016) of the isolates exhibited resistance to FLU (MIC ≥ 8 mg/L; Table 1). Notably, half of the FLU-R isolates (55.7%; *n* = 29/52) were from BSIs with resistance occurring in 4% cases of candidemia. Of the remaining FLU-R isolates, 32.6% were from urine, and the remaining 11.7% were from fluids and tissues. The yearly resistance rates ranged from 1.7% to 9.1% during 2014–2022 with the highest rate observed in the year 2021 (9.1%). Furthermore, 55.7% (*n* = 29/52) of FLU-R isolates displayed cross-resistance to voriconazole (VRC; MIC ≥ 1 mg/L), and 44.2% (*n* = 23/52) had elevated MICs (0.5–16 mg/L) for itraconazole (ITC). Of these 52 isolates, 36.5% were multiazole-resistant (FLU + VRC + ITC). Furthermore, low geometric mean-MIC (GM-MIC; 0.02 mg/L) was observed for isavuconazole (ISA) and posaconazole (POS). Three isolates with a MIC value of 1 mg/L for POS and two isolates with a high MIC value of ISA (1 and 4 mg/L) showed cross-resistance to both ITC and VRC. Low GM-MICs were observed for all tested echinocandins, amphotericin B (AmB), and 5-flucytosine (5-FC). Interestingly, 1.6% (*n* = 17) of clinical isolates exhibited high 5-FC MICs (0.5–64 mg/L). In contrast, all the environmental isolates were found to be susceptible to all tested antifungal agents.

**TABLE 1:** *In vitro* antifungal susceptibility profile of 1,016 clinical *Candida tropicalis* isolates and 16 environmental isolates against nine antifungal drugs using CLSI broth microdilution method

	Antifungal^*a*^	MIC^*b*^ (mg/L)															Range	GM-MIC^*c*^	MIC_50_^*d*^	MIC_90_^*e*^
		0.01	0.03	0.06	0.12	0.25	0.5	1	2	4	8	16	32	64	128	≥256				
Clinical isolates	FLU						764	104	85	11	10	5	8	12	8	9	0.5–≥256	0.77	0.5	2
	VRC		763	119	52	34	15	8	10	10	3	2					0.03–16	0.04	0.03	0.12
	ITC		548	189	145	69	34	25		2		4					0.03–16	0.05	0.03	0.25
	ISA	760	91	70	49	26	18	1		1							0.01–4	0.02	0.01	0.06
	POS	611	182	103	63	35	19	3									0.01–1	0.02	0.01	0.12
	AmB		68	68	221	273	312	64	10								0.03–2	0.23	0.25	0.5
	MFG	520	64	246	144	35	7										0.01–0.5	0.03	0.03	0.12
	AFG	507	72	58	174	184	21										0.01–0.5	0.04	0.03	0.25
	5-FC				811	7	3	1	1	2	1		4	5			0.12–64	0.13	0.12	0.12
Environmental isolates	FLU					12	3	1									0.25–1	0.31	0.25	0.5
	VRC		16														0.03	0.03	0.03	0.03
	ITC		16														0.03	0.03	0.03	0.03
	ISA		16														0.03	0.03	0.03	0.03
	POS		16														0.03	0.03	0.03	0.03
	AmB			1	3	12											0.06–0.25	0.20	0.25	0.25
	MFG		16														0.03	0.03	0.03	0.03
	AFG		16														0.03	0.03	0.03	0.03
	5-FC				16												0.12	0.12	0.12	0.12

^*a*^ FLU, fluconazole; VRC, voriconazole; ITC, itraconazole; ISA, isavuconazole; POS, posaconazole; AmB, amphotericin B; MFG, micafungin; AFG, anidulafungin; 5-FC, 5-flucytosine.

^*b*^ Fluconazole-resistant *C. tropicalis* isolates are indicated with underlined numbers.

^*c*^ GM-MIC, geometric mean minimum inhibitory concentration.

^*d*^ MIC_50_, MIC at which growth of 50% tested isolates was inhibited.

^*e*^ MIC_90_, MIC at which growth of 90% tested isolates was inhibited.

Target sequencing of the *ERG11* gene done on 102 clinical isolates (52 FLU-R and 50 FLU-susceptible; FLU-S MIC range 0.5–2 mg/L) showed that all FLU-R isolates carried well-recognized nucleotide mutation A395T/W which led to amino acid substitution Y132F in the azole antifungal target Erg11. In addition, 88.5% (*n* = 46) of resistant isolates with Y132F carried polymorphism S154F; the latter was not detected independently in any isolate tested.

### MLST clade 4 dominates azole-resistant Indian *Candida tropicalis* isolates and exhibits geographical association across Asia

**Fig 1:**
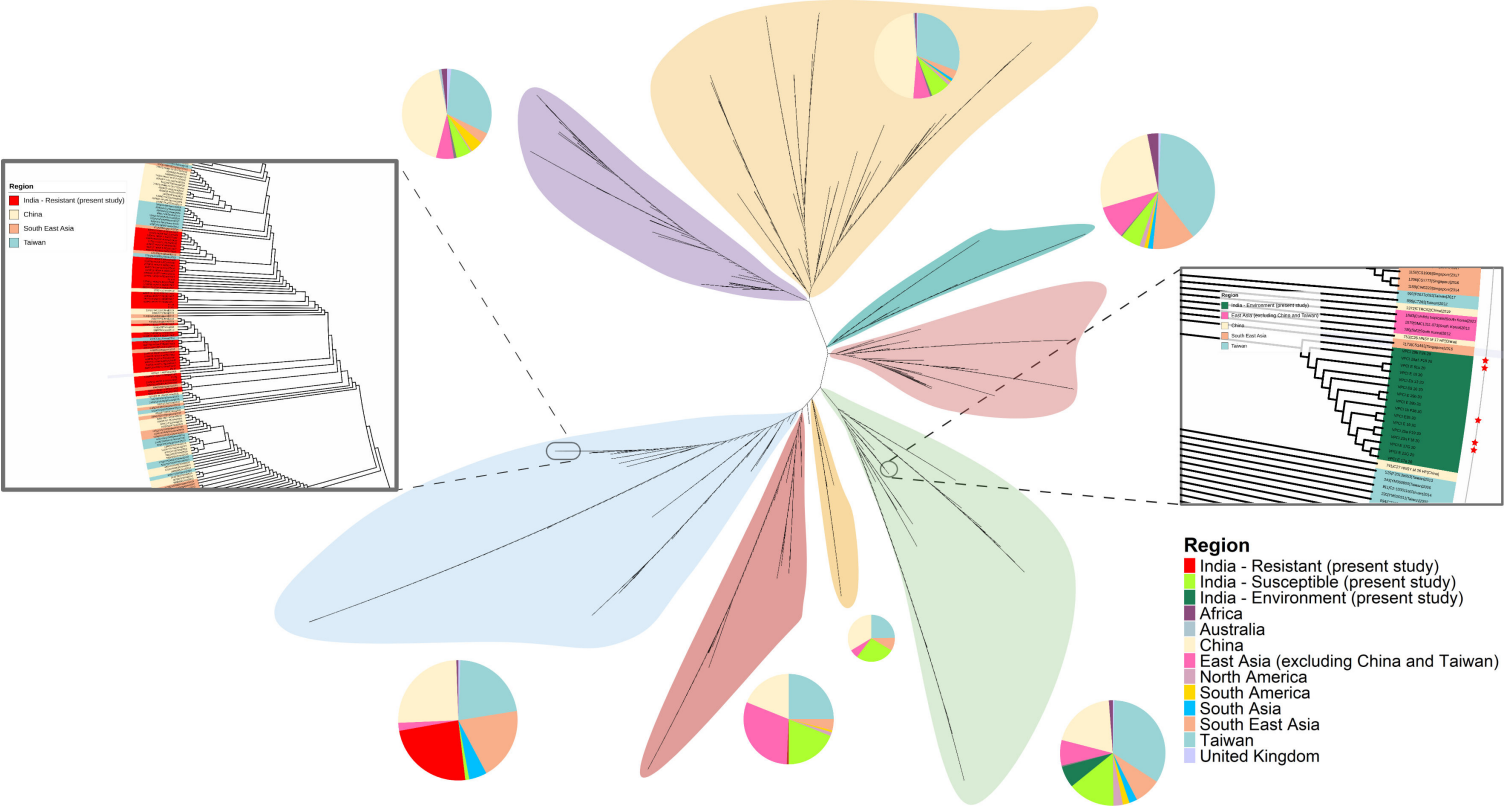
Unrooted maximum-likelihood (ML) tree based on concatenated sequences of six multilocus sequence typing (MLST) gene loci of 1,630 global *Candida tropicalis* isolates. Major phylogenetic branches are highlighted in different colors, and pie charts adjacent to each major branch depict the relative proportion of isolates from distinct geographic regions, with color codes corresponding to the legend shown at the bottom right. Azole-resistant (*n =* 45 clinical isolates, highlighted in red, are presented in the left inset panel to show the finer resolution of the branch, displaying close association with MLST clade 4 isolates belonging to China, Taiwan, and Singapore. Similarly, environment (*n =* 16) isolates are displayed on the right inset panel, and * mark represented the isolates from the surfaces of fruits. Geographic origins are represented by distinct colors throughout the tree.

We analyzed the MLST profiles of 1,630 isolates, including 1,422 global isolates retrieved from the PubMLST database and 208 isolates from the present study with 192 clinical (45 AZO-R and 147 azole-susceptible; AZO-S) and 16 environmental isolates collected previously from surfaces of fruit and hospital inanimate environment of a single ICU in Delhi. Overall, 1,630 isolates analyzed are detailed in the Table S1. A total of 112 diploid sequence types (DSTs) were identified among 208 isolates (Table S1). All AZO-R Indian clinical isolates belonged to MLST clade 4, as described previously in a global collection of 1,571 *C. tropicalis* isolates analyzed by Fan et al. (9) (Fig. 1). Interestingly, 75.5% of clade 4 isolates were multiazole-resistant, and 62.2% (*n* = 28/45) exhibited cross-resistance to VRC. The phylogenetic tree of global 1,630 isolates showed that all Indian AZO-R isolates clustered closely with AZO-R isolates from China, Singapore, and Taiwan, representing similar DSTs. Overall, isolates from North America, South America, Africa, and Europe fell separately, showing distinct geographic distribution (Fig. 1). The geographical clustering of Indian AZO-R *C. tropicalis* isolates in MLST clade 4 underscores the expansion of this clade across Asia.

### Prolonged persistence and transmission of azole-resistant clonal complexes in the hospitals

A wide range of DSTs (*n* = 96) listed in the *C. tropicalis* MLST database were identified in the clinical samples examined in the present study. Furthermoe, 12 (12.3%) newly identified DSTs which are not described in MLST database were detected in clinical samples. Interestingly, 55.5% AZO-R isolates harbored two DSTs, that is, DST542 (29%; *n* = 13/45) and DST539 (26.6%; *n* = 12/45). These two DSTs were observed predominantly in BSIs (*n* = 15) followed by urine (*n* = 6) samples. The remaining 20 AZO-R isolates exhibited DST538 (*n* = 8), followed by DST756 (*n* = 3) and DST1221 (*n* = 2) and are detailed in Table S1. Widely diverse DSTs (*n* = 84) were recorded in 147 susceptible isolates, including 12 novel DSTs (Table S1). Furthermore, all of the environmental isolates formed distinct DSTs clustering away from the Indian clinical and global isolates (Fig. 1; Table S1). The list of all DSTs identified is available in Table S1.

Furthermore, goeBURST analysis yielded eight clonal complexes (CCs) which were defined as sets of DSTs that share at least five or six alleles (single-locus variants). Overall, 8 CCs from CC1 and numbered in descending order based on the number of isolates within each CC and 78 singletons were identified (Table S1). Notably, 93.3% of the AZO-R isolates belonging to clade 4 were classified in CC1. Of the three remaining AZO-R isolates, 1 clustered with 29 susceptible isolates in CC2, while the other 2 were singletons. The remaining six complexes (CC3–8) consisted of 13, 7, 5, 3, 4, and 4 susceptible isolates, respectively (Table S1). Overall, more than one DST was detected in all hospitals with AZO-R CC1 circulating in 10 hospitals during the 7-year study period. Interestingly, between 2 and 12 cases due to AZO-R CC1 were associated with infections in a single hospital. For example, in Hospital A, 12 cases of BSIs belonging to CC1 were identified over a 6-year period (2015–2020). Furthermore, of these 12 CC1 isolates, 8 isolates were analyzed by WGS and showed a tight cluster representing 24–284 single-nucleotide polymorphisms (SNPs). Similarly, in Hospitals E and B, CC1 isolates (9 and 8, respectively) were detected over a 7-year span. Furthermore, phylogenomic analysis also closely clustered these isolates from E and B hospitals with SNPs ranging 14–342. Interestingly, MLST clade 4 isolates (CC1) circulating in the above three hospitals showed close genetic relatedness (12–343 SNPs) among them, indicating interhospital transmission of strains. Importantly, AZO-R isolates demonstrated prolonged persistence and clonal transmission within hospital settings over a long duration.

### Phylogenomic analysis showed Indian azole-resistant isolates cluster distinctly but close to the isolates from China

**Fig 2:**
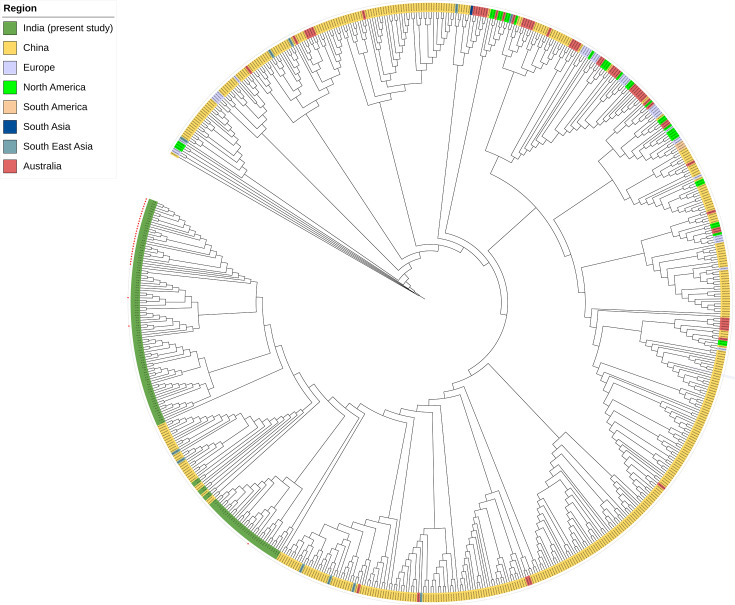
Whole-genome phylogeny depicting the relationships among 716 *Candida tropicalis* clinical isolates across the globe. Relationships were inferred based on their whole-genome single-nucleotide polymorphisms (SNPs). Colored labels indicate the geographic region for each isolate. Azole-resistant isolates from the present study are highlighted with a red star.

**Fig 3:**
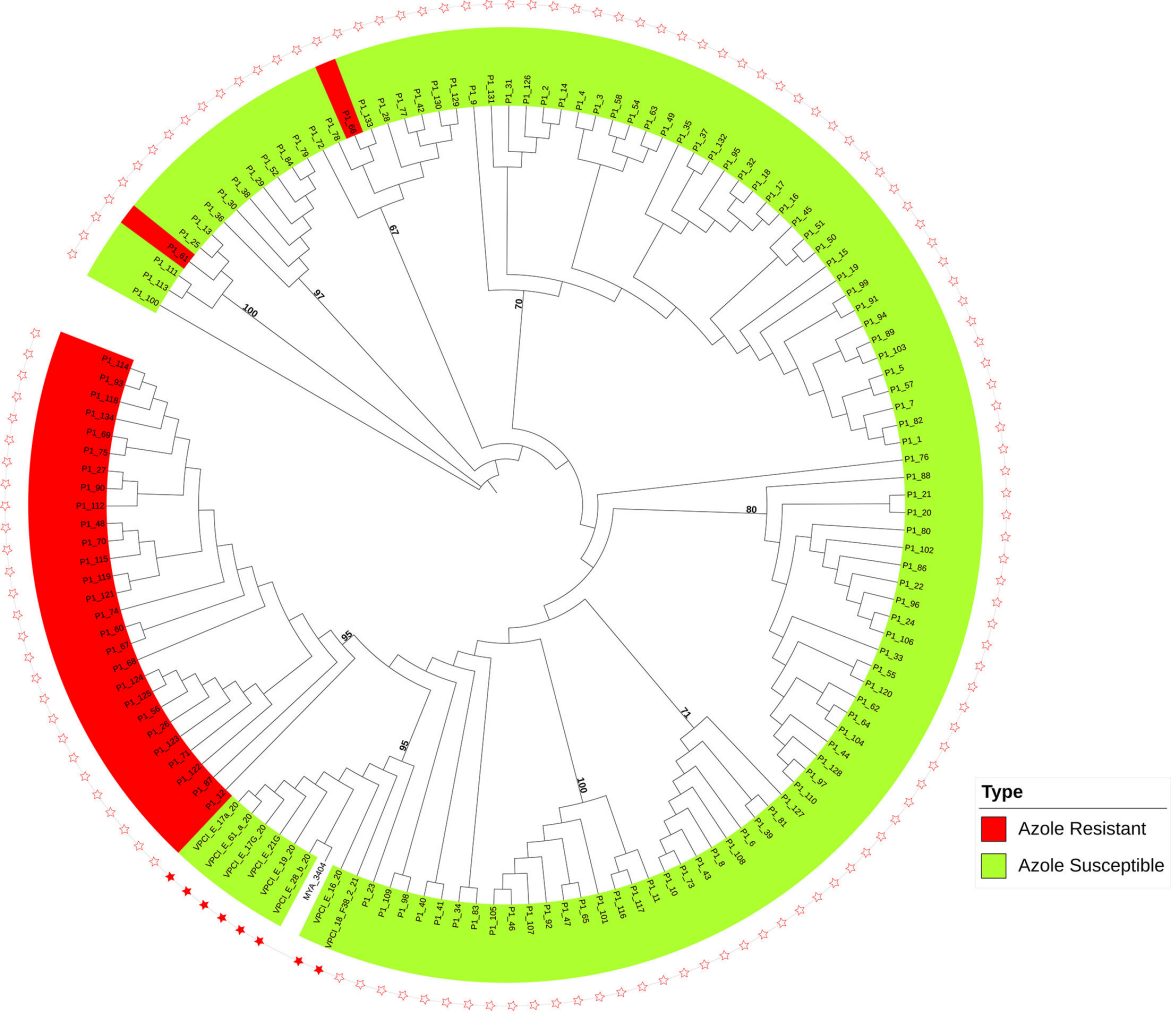
Maximum-likelihood phylogenomic tree of 139 *Candida tropicalis* strains included 131 clinical and 8 environmental isolates of the present study, constructed by using IQ-TREE2, along with the reference strain, MYA-3404. Azole-resistant isolates are labeled in red, while susceptible isolates are in green. Inanimate environment and isolates from the fruit surface are represented with a red-filled star, while unfilled stars indicate clinical isolates. Branch supports were assessed using 1,000 bootstrap replicates.

We investigated the phylogenomic relationship of 585 previously published genomes of *C. tropicalis* from 14 countries and 131 clinical isolates from this study. The details of 716 *C. tropicalis* isolates are provided in Table S2. Interestingly, all 131 clinical isolates formed a distinct cluster lying closest to isolates from China (324–58,231 single-nucleotide polymorphism; SNPs; Fig. 2). Furthermore, Indian clinical and environmental isolates showed large SNP differences (range 22,084–35,802; Fig. 3). Overall, AZO-R isolates formed a tight cluster positioned farthest from the root of the phylogenetic tree, indicating that these isolates have undergone significant evolutionary changes in the recent past. Notably, 90% of AZO-R isolates exhibited a close genetic relatedness, with SNP differences of 12 to 1,236 among them (Table S3). A genetically diverse population of susceptible isolates was observed forming 15 small clusters (2–14 isolates), with SNP differences ranging from 13 to 10,270 (Fig. 3). Principal component analysis (PCA) with 15.29% variance at PC1 also positioned all AZO-R strains separately from the AZO-S isolates. This separation indicates that the AZO-R isolates may follow distinct evolutionary trajectories, potentially facilitating their dissemination (Fig. S1). Environmental strains fell into two small clusters: Cluster A, comprising four genetically related isolates from the inanimate environment of a single hospital (74–130 SNP differences) and Cluster B, consisting of two related isolates, one from the inanimate environment and the other from a fruit surface (269 SNP differences) suggesting transmission of natural environment strains in the hospital settings (Fig. 3, Table S3).

### Loss of heterozygosity events and heterozygous mating locus in azole-resistant isolates

Heterozygosity percentages were inferred using reference genome GCA_013177555.1, consisting of seven contigs as described by Guin et al. (28). Most isolates possess an average of 2.00–6.34 heterozygous variants (SNPs and indels) per kilobase pair (kbp), with a mean of 4.23 heterozygous and 2.68 homozygous variants per kbp, consistent with previous reports (29–32). Overall, genome-wide heterozygosity was observed in 1.5% of all isolates. All loss of heterozygosity (LOH) blocks are a minimum of 100 bp in length. Average LOH block coverage across all isolates is 69.3% of the genome, with the average sum length of 10.11 Mb in each isolate. The largest LOH block length is 59.6 kbs in chromosome R. LOH events were observed in chromosomes 1–3, 5, 6, and R in both susceptible and resistant isolates. Interestingly, chromosome 5, which carries the *ERG11* gene, showed a 900 bp LOH block at position 1094487-1095386 in 60% (*n* = 18) of FLU-R isolates (Fig. S2a). Of these, 16 isolates were multiazole-resistant.

Overall, similar LOH patterns were observed among isolates from the same hospital during a specific time period (Fig. S2b). For instance, in Hospital I (year 2016), isolates showed similar LOH events at chromosome 3, and in Hospital S (year 2021), similar LOH patterns encompassing 45% of chromosome 6 and the terminal end of chromosome 2 were observed. Furthermore, in Hospital G (years 2021 and 2022), significant LOH events in the latter halves of chromosomes 2 and 6 were recorded. Similarly, inanimate environmental isolates (SNP 74-130) from a single hospital exhibited heterogeneity with large LOH blocks in chromosomes R and 1. Furthermore, as reported previously, all isolates were heterozygous consisting of both the MATa and MATα alleles at their mating-type (MAT) locus (29) (Table S2).

### Mutations, along with an increase in copy number and expression of the *ERG11* gene, are linked to multiazole resistance in *Candida tropicalis*

**Fig 4:**
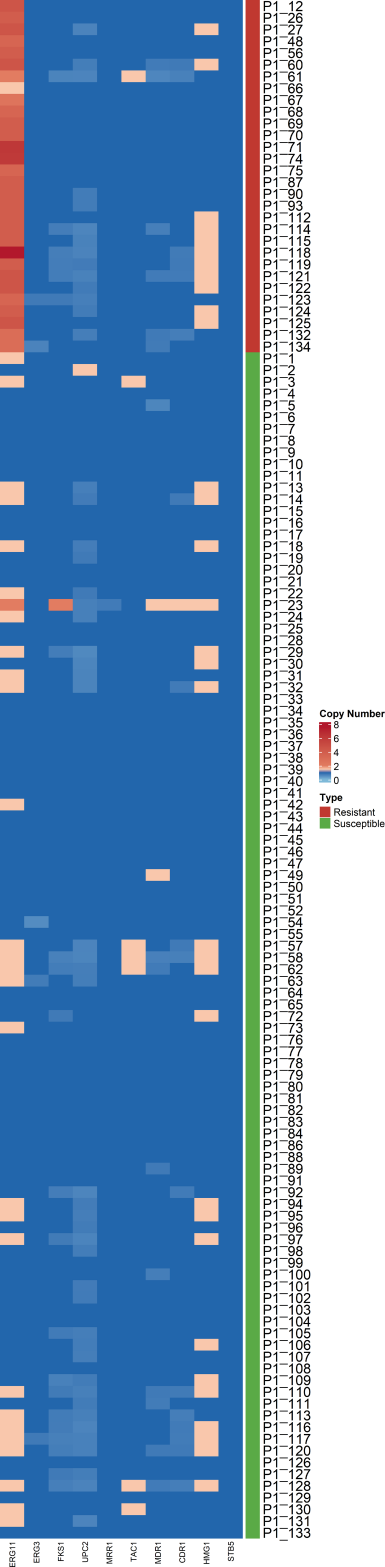
Heat map showing copy number variation in 30 azole-resistant (red panel) and 101 azole-susceptible (green panel) *Candida tropicalis* isolates across 10 selected antifungal resistance genes. Each row represents an isolate, and each column corresponds to antifungal resistance genes. Variations in color intensity indicate changes in copy number, with blue shades representing allele loss (decrease in copy number) and red shades representing allele gain (increase in copy number).

Screening of missense mutations across 33 drug resistance genes identified 841 polymorphisms and 13 mutations in clinical isolates, including the known nucleotide mutation A395T/W in the *ERG11* gene (Table S4). *ERG11* gene duplication was observed in 28 of the 30 AZO-R isolates (Table S5). Of these 28 AZO-R isolates, 26 showed multiazole resistance. Copy number variation (CNV) analysis showed that all AZO-R isolates except for a single isolate had high copy numbers ranging 2–7.5 of *ERG11* gene, implying that the resistant isolates possessed more than two alleles of *ERG11* gene (Fig. 4). Fluorescence-activated cell sorting (FACS) revealed diploid genome content in all AZO-R isolates (Fig. S3), with no evidence of aneuploidy as also detected by the SAM tools indicating that gene-level amplification, rather than aneuploidy, is the predominant mechanism for AZO-R. We investigated mRNA expression of the *ERG11* gene in 30 *C. tropicalis* isolates, including 17 AZO-R and 13 susceptible isolates. Real-time quantitative reverse transcription PCR (qRT-PCR) assays indicated that all AZO-R isolates showed significant overexpression of the *ERG11* gene with a fold change of 2.0–10.9 ± 0.77 (*P*-value < 0.05) as compared to the average of 13 susceptible isolates (Fig. S4). Indeed, 70.5% of AZO-R isolates (*n* = 12) showed sixfold higher expression of *ERG11* than susceptible isolates.

Among the AZO-R isolates, 90% (27/30) harbored missense mutations, including A446E in the transcriptional activator *TAC1* and L168P in the transcription factor *UPC2* (Table S4). Furthermore, the m-RNA transcript level of *CDR1* was upregulated in 41.1% (2–9.2 ± 0.39-fold; *P*-value < 0.05) of AZO-R isolates, whereas *UPC2* was not significantly overexpressed in 88.2% of the AZO-R isolates, suggesting that *CDR1* might also be contributing to azole resistance (Fig. S5).

### Transcriptional upregulation of virulence-associated genes and increased biofilm-associated metabolic activity in fluconazole-resistant strain

We compared genome-wide transcriptional profiles of FLU-R (P1_122; MIC > 256 mg/L) and susceptible (P1_128; MIC 0.25 mg/L) isolates exhibiting >512-fold difference in FLU susceptibility. A multivariate PCA revealed similarities between biological replicates and differences among the *C. tropicalis* isolates. The biological replicates from each isolate clustered together, underscoring high data correlation. The first two principal components separated the FLU-S and FLU-R isolates, explaining 69% of the variance. Differential expression analysis revealed 1,642 genes with altered expression in the FLU-R strain compared to the FLU-S strain. Among these, 968 genes were significantly upregulated, while 674 genes were significantly downregulated (Table S6). The expression analysis of both *ERG11* (CTRG_05283) and *CDR1* (CTRG_03159) was upregulated in the P1_122 strain, as assessed by qRT-PCR (Fig. S6).

Importantly, the pathway analysis of differentially expressed genes (DEGs) using the gene ontology analysis reveals a significant association between drug resistance and cellular pathway changes. Screening of virulence-associated genes showed significant upregulation of seven genes in the FLU-R isolate. The *ALS7* (CTRG_03787) gene within the agglutinin-like sequence (ALS) family that is involved in cell adhesion was significantly upregulated in FLU-R isolates (Fig. S6). In concordance with the overexpression of adhesion-related genes, the quantification of biofilm activity by using tetrazolium reduction assay showed significant increase in the biofilm-associated metabolic activity in the FLU-R isolate as compared to the FLU-S isolate (8.7-fold; *P*-value < 0.05; Fig. S8). Furthermore, disinfectant inhibition activity against biofilm of both FLU-R and FLU-S isolates showed a ≥80% reduction following exposure to all tested disinfectants (*P* < 0.05), except for the FLU-R isolate treated with hydrogen peroxide, which showed markedly reduced susceptibility (<50%).

In addition, two secreted aspartyl proteinases (SAP) genes, that is, *SAP7* and *SAP9*, were upregulated in FLU-R isolates (Fig. S6). Furthermore, four essential genes, that is, glycosylphosphatidylinositol (GPI)-anchored proteins encoding gene *IFF6* (CTRG_05838), *PGA13* (CTRG_03085), *SKN2* (CTRG_02540), and *FMP45* (CTRG_05042) related to cell wall organization that affect adhesion, virulence, and pathogenesis in *Candida* species were significantly upregulated in the resistant isolate (Fig. S6). Furthermore, genes induced due to stress, that is, *DDR48* involved in DNA Damage Response (CTRG_01916) and *YMX6* (CTRG_02112), were upregulated in P1_122 (Fig. S6). These findings provide valuable insights into the transcriptional adaptations of FLU-R *C. tropicalis*, highlighting the interplay between antifungal resistance and virulence mechanisms.

### Ergosterol alteration and metabolic differences in FLU-R strain

The comparative analysis of sterol profiles revealed a significant alteration in ergosterol content between the two strains. The FLU-S strain (P1_128) exhibited a ~2-fold reduction in ergosterol levels compared to the FLU-R strain (P1_122; *P*-value = 0.02) (Fig. S7). Furthermore, non-targeted metabolomic analysis of intermediates in the ergosterol biosynthesis pathway did not reveal any specific differences between the two isolates. However, a total of 15 differential metabolites were identified, comprising 12 upregulated and 3 downregulated metabolites. KEGG pathway enrichment analysis showed that these metabolites were significantly enriched across 11 metabolic pathways (Table S7). Notably, among the differential metabolites, the saturated fatty acid heptadecanoic acid was significantly upregulated (6.9-fold), whereas the unsaturated fatty acid (6Z)−13-methyl-6-pentadecenoic acid was downregulated (~3-fold) in the FLU-R isolate (Table S7), indicating increased membrane saturation. Collectively, the elevated ergosterol content and increased proportion of saturated fatty acids in the FLU-R strain are likely to reduce membrane fluidity, thereby enhancing tolerance to drug-induced stress.

### Attenuated β-glucan exposure enhances immune evasion in fluconazole-resistant *Candida tropicalis*

**Fig 5:**
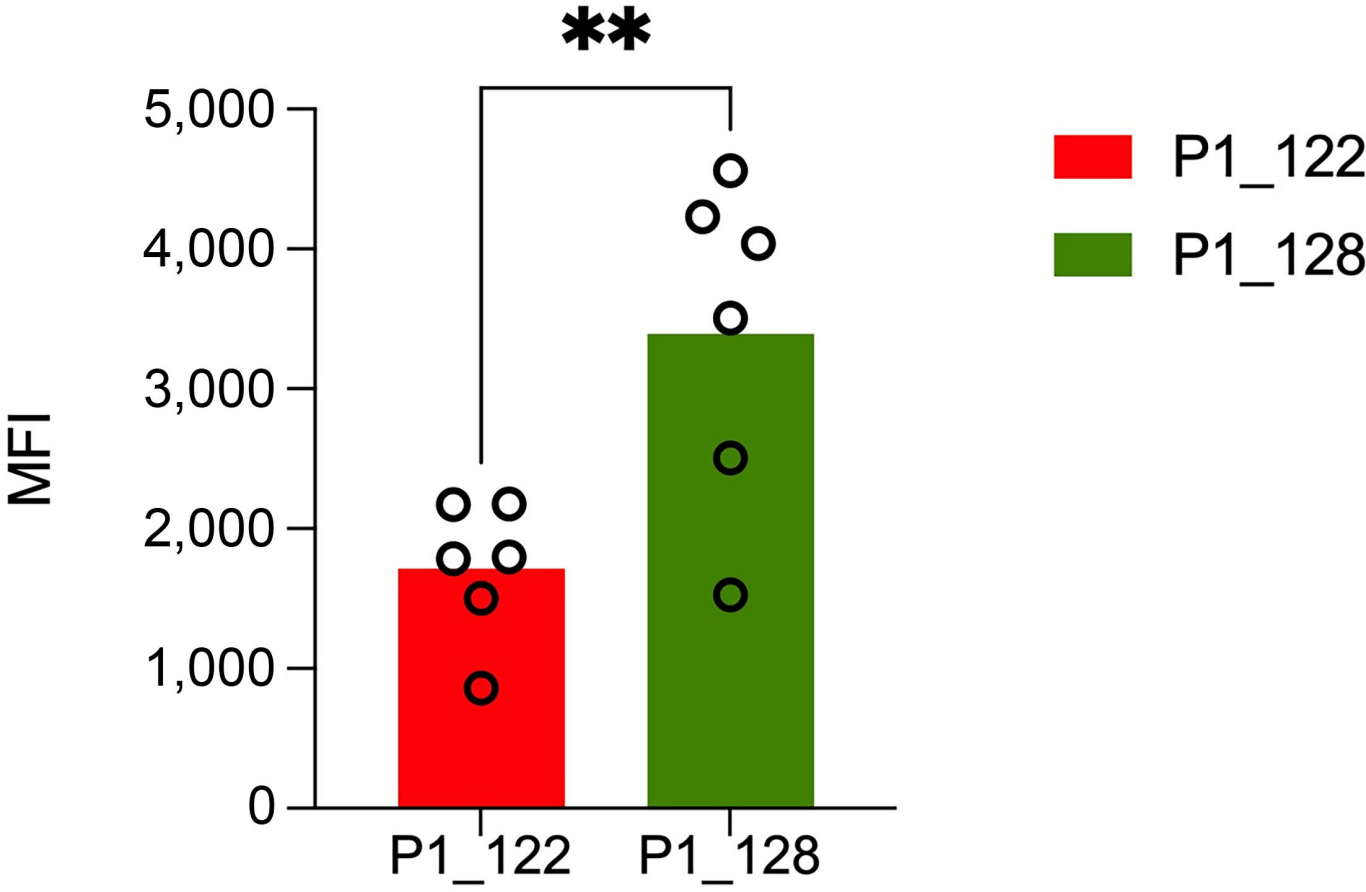
The bar graph depicts the β-glucan content in a fluconazole-resistant isolate (P1_122; red) and a fluconazole-susceptible isolate (P1_128; green). The *Y*-axis represents the mean fluorescence intensity (MFI), indicating glucan levels. Error bars represent standard deviation, and data points show individual replicates. The double asterisk (**) denotes a statistically significant difference between the groups (*P* < 0.01).

The differential expression of genes involved in cell wall organization in the FLU-R isolate, considering that the fungal cell wall is the first point of contact during interaction with host phagocytic cells, suggests that these changes may play a crucial role in host-pathogen interactions. To assess differences in fungal cell wall composition between the FLU-R (P1_122) and susceptible (P_128) *C. tropicalis* isolates, the same isolates as used for transcriptomic profiling, we quantified key carbohydrate components of the fungal cell wall, β-glucan, mannan, and chitin, by using a flow cytometry-based assay (33). Our results revealed significantly higher β-glucan levels in the FLU-S isolate compared to the FLU-R strain, indicating increased cell surface exposure of β-glucan (*P* < 0.01; Fig. 5). In contrast, mannan and chitin levels remain unchanged between the two isolates (data not shown). These results suggest that the FLU-R strain P1_122 may mask β-glucan more effectively, potentially contributing to enhanced immune evasion by limiting the recognition by host pattern recognition receptor (PRR) such as Dectin-1. Reduced β-glucan exposure in the FLU-R isolate is likely mediated through cell wall remodeling as transcriptomic analysis also revealed differential expression of multiple genes involved in cell wall organization and remodeling in the FLU-R isolate compared to the susceptible strain.

**Fig 6:**
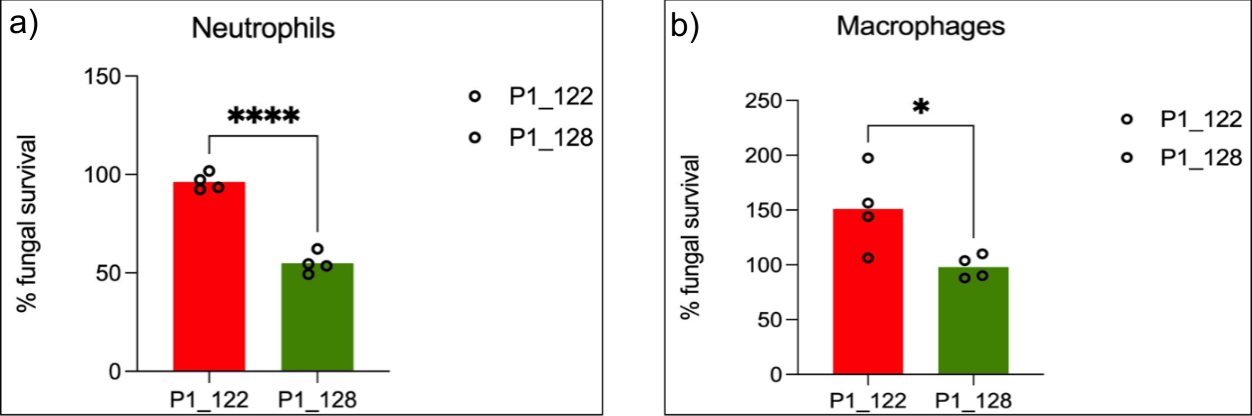
The bar graph represents the survival percentage of *Candida tropicalis* for fluconazole-resistant isolate (P1_122; red) and fluconazole-susceptible isolate (P1_128; green) (**a**) after neutrophil exposure and (**b**) following macrophage exposure. Error bars indicate standard deviation, and individual data points are shown. The reduction in fungal survival for P1_128 indicates greater susceptibility to neutrophil and macrophage-mediated killing. Statistical significance: ****, *P* < 0.0001; *, P < 0.05.

Macrophages and neutrophils, the primary myeloid phagocytes, play a central role in innate immune defense against fungal pathogens by employing both oxidative and non-oxidative mechanisms to eliminate fungal cells (34). To investigate whether FLU resistance in *C. tropicalis* is associated with altered susceptibility to immune-mediated killing, we co-cultured FLU-R (P1_122) and FLU-S (P1_128) clinical isolates with differentiated human neutrophil-like (PLB-985) and macrophage-like (THP-1) cells at a multiplicity of infection (MOI) of 1. Fungal survival was quantified after co-culture using the 2,3-bis(2-methoxy-4-nitro-5-sulfophenyl)−5-[(phenylamino)carbonyl]−2H-tetrazolium hydroxide (XTT) assay. Strikingly, the FLU-R isolate demonstrated significantly higher survival compared to P1_128 when incubated with neutrophils (Fig. 6a), indicating a marked resistance to neutrophil-mediated killing. A similar pattern was observed in macrophage co-culture assays, although the magnitude of difference was less pronounced (Fig. 6b). These results suggest that FLU resistance in *C. tropicalis* may be associated with reduced vulnerability to innate immune responses.

## DISCUSSION

We report the emergence and spread of AZO-R *C. tropicalis* clonal lineage of MLST clade 4 in several hospitals in Delhi and associated regions in North India over the last decade. Furthermore, this study showed that the lineage is associated with multiazole resistance (FLU and VRC) and displays immune evasion traits. Notably, our findings revealed that FLU-R MLST clade 4 *C. tropicalis* isolates were characterized by robust biofilm formation *in vitro* and showed resistance to being cleared by neutrophils and macrophages, the key components of the host immune system. Previously, WGS and MLST mapped the occurrence of this dominant lineage (MLST clade 4) among clinical samples in China in the year 2010 (9). The present study identified the first isolate in 2015 within our clinical isolates collection and subsequently spread in several hospitals in less than a decade. Apart from China, the expansion of this lineage in other Asian countries (Singapore and Thailand) and Australia has been reported in a limited number of isolates (9). Based on phylogenomic analysis, the Indian AZO-R lineage was closely related to AZO-R isolates from mainland China and Taiwan, although the direction of the intracontinental transmission within Asia is unknown and could signal the beginning of a greater spread of this clone. Furthermore, in addition to antifungal resistance, the isolates of this lineage exhibit several traits associated with adhesion and immune evasion that could possibly enable its spread in healthcare settings, leading to a public health concern scenario in time ahead. Also, global transmission potential due to widespread migration as seen previously with *C. auris* and *C. parapsilosis* remains a matter of concern (35, 36).

The underlying mechanism of azole resistance in the Indian AZO-R clone is primarily associated with key A395T/W mutations and gene duplication in *ERG11* gene coupled with copy number variations and elevation in the expression of the azole target gene. The study reports for the first time the expansion of the highly AZO-R MLST clade 4 clones outside mainland China, Taiwan, Singapore, Australia, and Thailand. Overall, we observed a 5% azole resistance rate spanning across 15 hospitals in a decade with presence of clade 4 isolates in 12 hospitals persisting during a 1- to 7-year period. Moreover, interhospital transmission linked to the close relatedness of isolates (12–343 SNPs) causing infections across several years involving three healthcare facilities indicates smoldering outbreak probably facilitated by transfer of patients between healthcare facilities and subsequent person-to-person transmission. In the present study, isolates from the inanimate hospital environment and from the fruit surface showed high genetic relatedness suggesting transmission of natural environment strains of *C. tropicalis* in the hospital environment. *C. tropicalis* outbreaks in ICUs have been associated with the contamination of hospital environments including hemodialysis machines from water and spread directly through contaminated equipment and via hands of healthcare workers (12, 37, 38). In the present study, the source of MLST clade 4 isolates in clinical settings remains speculative due to the limited number of environment samples tested. *C. tropicalis* exists in wide environmental niches such as soil, water, fruits, plants, and animals (13). The azole resistance in *C. tropicalis* has been linked to extensive clinical use of azoles in humans and use of agricultural azole fungicides in Taiwan and mainland China (13, 39). MLST clade 4 strains of *C. tropicalis* have been identified in environmental sources in China, from fruits purchased at a supermarket in northern Taiwan and from the environmental samples of orchards (9, 40). Previously, *C. tropicalis* has been isolated from the surfaces of fruits collected from fruit sellers in Delhi, India (41). Also, AZO-R *C. tropicalis* isolates have been identified in the gastrointestinal tract of poultry from India; however, the genetic characterization of the isolates was lacking (42).

In the Asia-Pacific region, a study from Japan reported 30.2% (16/53) of the *C. tropicalis* isolates to be AZO-R, with high levels of azole resistance among clinical isolates (51.7%; 15/29) and low levels (4.2%; 1/24) among environmental isolates (43). Furthermore, MLST showed multiple sequence types, and neither *ERG11* mutation nor overexpression was associated with azole resistance (43). The absence of clonal transmission in Japan is in contrast with the scenario in India and China, suggesting that AZO-R clonal strains have emerged and expanded in the two highly populated regions of the world. Furthermore, it is important to emphasize that the spread of infections caused by this drug-resistant yeast is a challenge to control, particularly in the regions with limited resources, high patient load, and limited availability of antifungal susceptibility testing facilities. Subsequently, this results in the transmission of the resistant strains to the vulnerable hosts through contaminated hands or equipment, specifically if the stringent infection prevention measures are not strictly adhered (37, 38). In contrast to the high FLU resistance rates reported among low-middle income countries in the Asia-Pacific and Latin America, data from high-income countries in Europe show marked reduction in susceptibility to 5-FC in a substantial proportion of *C. tropicalis* isolates >30% (27). Recently, the rapid emergence of a 5-FC-resistant, non-wild-type (WT) *C. tropicalis* clade, accounting for >40% of all *C. tropicalis* isolates was detected in the Netherlands (27). In the present study, we found that 1.6% of the isolates were 5-FC non-WT and no cross-resistance to other antifungal agents was observed.

Although worldwide studies focusing on the attributable mortality rates due to *C. tropicalis* invasive diseases have been lacking, a pan-European multi-centric, observational study reported candidemia-related mortality on a species-specific basis (4). The highest species-dependent attributable mortality, that is, 63.6% was observed for *C. tropicalis*, followed by *N. glabratus* (4). The formation of biofilm and the ability to adhere to host surfaces are important virulence factors of *Candida* species. Notably, *C. tropicalis* is characterized by its strong ability to form biofilms compared to other *Candida* species (15, 44). Recent studies have demonstrated that biofilm-producing *C. tropicalis* isolates have been associated with high mortality rates in patients with candidemia (45, 46). Importantly, infections associated with biofilm-forming isolates were linked to a significantly higher mortality rate (70.0%) compared to those caused by planktonic cells (37.9%) (47). A recent study demonstrated that MIC values of FLU were elevated in 87.5% (7/8) of *C. tropicalis* biofilm-forming isolates as compared to their planktonic counterparts (48). Furthermore, statistical analysis revealed that particularly in bloodstream isolates, FLU resistance was markedly increased in *C. tropicalis* strains exhibiting moderate biofilm-forming ability (100%) and in *C. albicans* strains categorized as high and moderate biofilm-formers (75%). In contrast, strains with low biofilm-forming ability, such as *C. parapsilosis* and *N. glabratus*, showed only a slight increase in resistance (<50%). These findings support the notion that enhanced biofilm-forming capacity in *Candida* species correlates with elevated resistance to FLU (48). Our study reinforces that FLU-R isolates produce abundant biofilm-associated adhesion as compared to the susceptible isolate, suggesting that the resistant isolates may effectively persist in the abiotic and biotic surfaces in the hospitals. However, we observed a marked reduction in biofilm metabolic activity following exposure to disinfectant, suggesting appropriate surface cleaning using commonly employed disinfectants may aid in infection control. In *Candida* spp., the adhesion ability is encoded by several gene families, the most important ALS gene family, which encodes large cell-surface glycoproteins that play a role in adherence of the yeast *C. albicans* to endothelial and epithelial cells (49). The most widely examined are the Als adhesins comprising eight members (Als1p–Als7p and Als9p), which are GPI-linked to β−1,6 glucans in the fungal cell wall. A *C. albicans* mutant unable to express *ALS3* forms weaker biofilms compared to the WT strain *in vitro*, and disruption of both *ALS3* and *ALS1* (*als3*ΔΔ/*als1*ΔΔ) reduces the biofilm biomass when compared with controls. Also, other Als family members (*ALS6*, *ALS7*, and *ALS9*) exhibit significant contributions to biofilm development as overexpression of these genes rescues the biofilm defect of an *als3*ΔΔ/*als*1ΔΔ mutant (49). Among the Als family, *ALS7* is one of the members, involved in the formation of biofilm in *C. tropicalis* (50). We observed a high expression of *ALS7* in the FLU-R isolate. Previously, a strong positive correlation between higher expression of the *ALS7* gene and adhesion to the extracellular matrix under shear forces has been reported in *C. parapsilosis* (51).

In addition to being a strong biofilm producer, the FLU-R *C. tropicalis* strain also demonstrated a greater ability to evade or survive phagocytic attack compared to the FLU-S strain. In humans, the innate immune response is activated in invasive fungal infection, where pathogen-associated molecular patterns (PAMPs) are recognized by PRRs triggering the activation of phagocytic cells to fight the infection (52). β-glucans, which are critical PAMPs, play a vital role in host–pathogen interactions (52). Recognized by the PRR Dectin-1, β-glucans serve as potent immunogenic signals. A previous report demonstrated that AmB-resistant *C. tropicalis* isolate exhibits an expanded cell wall with elevated levels of β−1,3 D-glucan (52). We observe that the FLU-R strain displays reduced β-glucan exposure, which may dampen host immune recognition and promote immune evasion. This is supported by our neutrophil and macrophage killing assays, in which FLU-S isolates were more efficiently cleared compared to FLU-R strains. A similar phenomenon has been described in *C. auris*, where alterations in cell wall components affect immune recognition and cytokine signaling (53). Our findings extend this concept to *C. tropicalis*, suggesting a convergent strategy among this species to evade innate immunity. Finally, epidemiological surveillance of the drug-resistant clades is of paramount importance as evidenced by the rapid expansion of AZO-R clade 4 strains in *C. tropicalis* warranting effective strategies to address the growing threat of antifungal resistance.

## MATERIALS AND METHODS

### Isolate collection and identification

During a period of 9 years (January 2014–December 2022), 1,016 *C. tropicalis* clinical isolates from 27 hospitals in Delhi and associated National Capital Region, which include adjacent districts of three states of India (Uttar Pradesh, Haryana, and Rajasthan), and 16 environmental isolates, including 11 from inanimate environment of a single hospital in Delhi and 5 strains from the surfaces of fruit previously published, were analyzed (41, 54). Isolates were identified by matrix-assisted laser desorption ionization–time of flight mass spectrometry (Bruker Biotyper OC version 3.1; Daltonics, Bremen, Germany) (55).

### Antifungal susceptibility testing

All isolates were subjected to antifungal susceptibility testing using the CLSI broth micro-dilution method following M-27, A3 (56). Antifungal susceptibility testing for nine antifungals was performed as described previously (55). The dilution ranges for nine antifungal drugs were as follows: 0.03–16 mg/L for ITC, VRC, and AmB; 0.01–8 mg/L for ISA, POS, and echinocandins; 0.5–256 mg/L for FLU; and 0.12–64 mg/L for 5-FC. FLU resistance in *C. tropicalis* was defined as MIC ≥ 8 mg/L, and FLU-S exhibited MIC of ≤2 mg/L (57). For VRC, and echinocandins, MIC of ≥1 mg/L was defined as resistant (57). In accordance with CLSI M57S 4th edition, isolates with MIC value of ≥0.5 mg/L for ITC were categorized as non-WT (58). Furthermore, no breakpoints for 5-FC have been assigned in this document; hence, isolates with a MIC value of ≥0.5 mg/L were categorized as non-WT for 5-FC according to previously published study (27). MIC values were determined visually. Furthermore, readings by spectrophotometer at 530 nm (Infinite 200 PRO microplate reader, Tecan, Männedorf, Switzerland) were taken to avoid misinterpretation of trailing as resistance. Isolates showing less than 49% growth were considered to exhibit trailing rather than true resistance (59).

### Azole target *ERG11* gene sequencing

Screening of *ERG11* gene was undertaken in 102 *C. tropicalis* isolates, including all 52 FLU-R (MIC ≥ 8 mg/L) and randomly selected 50 FLU-S (MIC 0.5–2 mg/L). DNA extraction and *ERG11* gene amplification were done as described previously by using primers detailed in Table S8 (55).

### Multilocus sequence typing using six housekeeping genes

Genetic relatedness was assessed using 208 *C. tropicalis* isolates, including 192 clinical isolates (45 AZO-R and 147 susceptible) from 19 hospitals selected to ensure temporal representation, along with 16 environmental isolates. Among the clinical isolates, conventional MLST gene sequencing was performed for the 102 isolates, while WGS-based MLST (wgMLST) profiles were retrieved for 90 isolates. Genotyping was done using six *ICL1*, *MDR1*, *SAPT2*, *SAPT4*, *XYR1*, and *ZWF1α* housekeeping genes as described previously (9). BioEdit software (version 7.0.5.3) was used to make consensus sequences. The nucleotide sequence’s identity was evaluated using BLASTN searches in the GenBank database, considering a sequence identity value of ≥99% for identification at the species level. Allelic profiles and the DSTs of the six gene sequences were obtained from the *C. tropicalis* MLST sequence-type database (http://pubmlst.org/ctropicalis/).

#### wgMLST

A combination of open-source software and custom scripts was used for wgMLST analysis. For five of the six loci (*ICL1*, *MDR1*, *SAPT2*, *SAPT4*, and *ZWF1a*), variants were directly extracted within the reference region of each loci from the multisample VCF and reconstructed using GATK FastaAlternateReferenceMaker. Similarly, as mentioned by Fan et al. (9), *XYR1* contained two copies of *XYR1* in the haploid chromosome set; therefore, we performed a separate read mapping process with a single copy of *XYR1* to identify variants and reconstruct the loci for each isolate. Finally, we identified allele numbers and DSTs using the pubMLST database (https://pubmlst.org).

### Multilocus sequence typing-based dendrogram of global *Candida tropicalis* isolates

Publicly available MLST data set (until 24 January 2025) was downloaded from PubMLST (https://pubmlst.org/), and all sequences of *C. tropicalis* originating in Asia and Oceania were included along with 20 isolates each from countries in North America, South America, Europe, and Africa. Overall, 1,630 isolates were analyzed, including 208 from the present study. The isolates were aligned using Multiple Alignment using Fast Fourier Transform (MAFFT) MAFFT v.7.520 (60). A maximum-likelihood (ML) tree using IQ-TREE v.2.2.5 was constructed, incorporating 1,000 ultrafast bootstrap replicates to assess branch support and phylogenetic reliability (61). To classify the isolates into MLST clades, we followed the established nomenclature defined by Fan et al. (9). Furthermore, clonal complexes (CCs) were analyzed using the goeBURST (62) algorithm, which were defined as sets of DSTs that share at least five or six alleles (single-locus variants) in PHILOViZ v.2.0 (63).

### Whole-genome sequencing

A total of 139 *C. tropicalis* isolates comprising 131 clinical (30 AZO-R and 101 AZO-S) and 8 environmental isolates were sequenced using a NOVASEQ 6000 sequencer (San Diego, USA). Clinical isolates were selected on the basis of their year of isolation and equal representation of varied types of clinical specimens. DNA extraction and quantification were done as described previously (55). In addition, raw FASTQ reads of the reference strain MYA-3404 were retrieved from NCBI. Furthermore, all 140 isolates were processed following a standard procedure of removal of adapters and low-quality reads using Trim Galore v.0.6.10 (https://github.com/FelixKrueger/TrimGalore). The filtered reads were mapped to the reference *C. tropicalis* MYA-3404 (RefSeq accession: GCF_000006335.3) using the Burrows-Wheeler Aligner v.0.7.17 (64). SAMtools v.1.18 was used to process the SAM files to convert and sort the BAM files by coordinate (65). Variant calling was performed using the Genome Analysis Toolkit v.4.0.5.1 (29). Finally, the variants were annotated using SnpEff v.4.3t (66). SNP trees were drawn using IQ-TREE v.2.2.5 by using the “GTR+FO + G4 m” model with 1,000 bootstrap value (67).

#### Phylogenetic analysis of global *Candida tropicalis* isolates

A total of 716 isolates, including 585 previously published genomes of *C. tropicalis* from 14 countries along with 131 clinical isolates of the present study, were analyzed to assess the genetic relatedness of Indian isolates with global data. We included both heterozygous and homozygous SNPs, while implementing a filtration criterion of depth >10 and removal of indels from the data set. We inferred an ML tree from 2,216,857 confident SNPs using IQ-TREE v.2.2.5, with the flags “-m GTR -mrate CAT” for a consistent model (68).

#### Principal component analysis

PCA was performed to assess genetic relationships among the isolates. Variant data were processed using PLINK v1.9.0, and PCA was computed based on genome-wide single-nucleotide polymorphisms. The resulting principal components were then visualized through an R script.

#### Mutation analysis, copy number variation, and gene duplication

SNPs in 33 genes related to antifungal resistance were identified using a combination of bcftools and custom R scripts.

To perform CNV analysis, BAM files were processed using the “Splint” script (69) to remove the “smiley pattern” bias observed at the terminal ends of chromosomes. The sequencing depth of each base was calculated using the SAMtools “depth” command. The mean read depth across non-overlapping windows of 500 bp was calculated and visualized using a custom R script (https://github.com/nityendra21/tropicalis_scripts). Coverage ratios for each window were determined as log2 (window coverage/mean whole genome coverage). The round-off method was used for CNV estimation of genes of interest, as previously described by Hu et al. (70). Gene duplication was assessed by summarizing window-based copy number estimates at the gene level. Annotated gene coordinates were intersected with the 500-bp windows, and log2 coverage ratios overlapping each gene were aggregated. Genes exhibiting consistently elevated log2 coverage ratios greater than two were classified as duplicated.

#### Loss of heterozygosity and mating type locus (MTL) analysis

JLOH v.1.0.2 (71) was used to calculate blocks of 100 bp across the genome as described previously (29). The SNP density was calculated using the JLOH “stats” function. LOH blocks were inferred using the JLOH “extract” function with parameters “--min-length 100 –min-snps-per-kbp 2,6”. Finally, the LOH blocks were plotted using the JLOH “plot” function.

Primers for MTLa and MTLα were retrieved from (72) and were used to extract complete sequences from the genome assembly and confirmed using BLAST. MTL gene coverage was calculated by mapping the sequences to raw reads and calculated by using SAMtools “depth.”

### Ploidy analysis of *Candida tropicalis* strains using FACS

All AZO-R clinical *C. tropicalis* isolates were subjected to FACS (FACSAria III; BD Biosciences, USA). The reference strain of *C. albicans* ATCC90028 was used as a diploid control. Sample preparation and analysis were done, as described previously (55). FlowJo_v10.10.0 software was used to generate the FACS layout diagram. Additionally, we checked for any possible ploidy events by plotting the normalized whole-genome depth of coverage data (9), called using the SAMtools “depth” command and custom R scripts for visualization.

### Real-time quantitative reverse transcription PCR assay

Expression levels of four important genes attributing to azole susceptibility in *Candida* species, including *ERG11*, its transcription factor *UPC2*, drug transporter genes *CDR1*, and *MDR1*, were analyzed in 30 *C. tropicalis* isolates on the basis of their FLU-MIC values, including 17 FLU-R isolates (FLU-MIC ranging from 8 to >256 mg/L) and 13 FLU-S isolates with FLU-MIC 0.5–2 mg/L. Culture for RNA extraction was prepared as described in (55). RNA was extracted using Maxwell RSC Plant RNA Kit (Promega, Madison, WI, USA) as recommended by the manufacturer. Purity of RNA, cDNA synthesis, and qRT-PCR preparation were performed according to Jain et al. (55) on the QuantStudio 5 (Applied Biosystems, Massachusetts, USA) (55). Table S8 details the primers used in the analysis (20, 73). The gene *ACT1* was used as an internal control. Fold changes in gene expression were determined using the 2^−ΔΔCT^ analysis method as described previously (55). The expression of target genes in FLU-R isolates (*n* = 17) was evaluated relative to the average of the FLU-S isolates (*n* = 13), and a *t*-test was applied to determine the significance level.

### RNA-seq of fluconazole-susceptible and fluconazole-resistant isolates

We performed comparative transcriptomic analysis of two *C. tropicalis* bloodstream isolates: a FLU-R MLST clade 4 isolate (P1_122; MIC >256 mg/L) and a FLU-S isolate (P1_128; MIC 0.25 mg/L), selected on the basis of their contrasting FLU-MICs, *ERG11* genotypes, and MLST backgrounds. Culture preparation, RNA extraction, and quantification were done as described above. The quality of RNA was checked using TapeStation (Agilent Technologies, Palo Alto, CA, USA). Following the manufacturer’s instructions, the RNA sequencing libraries were prepared using the NEBNext Ultra II RNA Library Preparation Kit (New England Biolabs, Ipswich, MA, USA). The samples were sequenced using a 2 × 150 bp paired-end configuration. Three biological replicates for each strain were sequenced. The raw FASTQ reads were quality controlled and aligned to the reference strain of MYA-3404 using HISAT2 with default settings (74). The mapped transcripts’ read counts were quantified using FeatureCounts (75). Differentially expressed genes were analyzed using DESeq2 (76). Genes with false discovery rate of ≤0.05 and absolute log2 fold changes of ≥0.585 (1.5-fold change) were called DEGs for each comparison. Both PCA and volcano plots were generated using the DESeq2. Furthermore, functional annotation of the gene accessions was done using Blast2GO (77), and enrichment analysis was performed with g:Profiler (78).

### Sterol extraction and quantitation

To assess differences in ergosterol content between FLU-R and FLU-S *C. tropicalis* strains, sterol extraction and quantification were performed on the same two isolates: one FLU-R (P1_122) and one FLU-S (P1_128). Both strains were grown overnight at 37°C with shaking at 200 rpm for 16 h, followed by subculturing in 50 mL YPD broth to an OD₆₀₀ of 1 and further incubation for 3 h at 37°C. Sterols were extracted using a 2:1 chloroform:methanol solvent system as described previously (79). Extracted samples were vacuum-dried and derivatized with O-bis(trimethylsilyl) trifluoroacetamide containing trimethylchlorosilane (BSTFA/TMS; Sigma-Aldrich, Darmstadt, Germany). Sterol analysis was performed using gas chromatography–mass spectrometry (QP2010 SE, Shimadzu, Japan), and data were analyzed using Xcalibur software (Thermo Scientific). A standard curve with standard ergosterol was generated and used to calculate the amount of ergosterol. Ergosterol levels were expressed as mean values obtained from three independent biological replicates (80).

### Metabolomic analysis

To assess differences in the level of metabolites, the same FLU-R and FLU-S *C. tropicalis* isolates selected for metabolomic analysis. Metabolite extraction was performed using the METPREP Kit (Advait Theragnostics Pvt. Ltd., Gujrat, India) following the manufacturer’s protocol. Following extraction, samples were analyzed using a high-resolution LC–MS platform comprising a Thermo Scientific Vanquish UHPLC quaternary system coupled to a Thermo Scientific Orbitrap Exploris 240 mass spectrometer, operated in both positive and negative ionization modes under a data-independent acquisition strategy. Chromatographic separation was achieved on a Thermo Hypersil GOLD C18 column (100 × 2.1 mm, 1.9 μm) maintained at 30°C, with a constant flow rate of 0.3 mL/min. Mobile phases were prepared using the METPREP Kit (Advait Theragnostics Pvt. Ltd.), according to the manufacturer’s specifications. The gradient was initiated from 0 to 30 min with decreasing percentage of Buffer B from 95% to 2% and subsequently re-equilibrated to 95% Buffer B for 5 min. Mass spectrometric data were acquired over an *m*/*z* range of 100–1,500. Raw LC–MS data were processed and annotated using Compound Discoverer software, followed by comprehensive statistical and multivariate analyses using the Metline 2.0 (AIML-based data analysis pipeline) for discovery metabolomics. This untargeted workflow was applied to metabolomics profiling of two *C. tropicalis* strains: susceptible vs resistant. Following feature curation, metabolites were classified into common and strain-specific (unique) metabolite sets, allowing comparative metabolic characterization across strains. Statistical analyses were selectively applied to the common metabolite pool to identify significantly altered metabolites and shared metabolic signatures.

### Biofilm metabolic activity quantification using tetrazolium reduction assay

We performed quantification of biofilm metabolic activity using tetrazolium reduction assay with the XTT for the same two isolates subjected to transcriptomic profiling. The XTT colorimetry assay was performed according to (81). Colorimetric change in the XTT reduction assay was measured in a microtiter plate reader (Infinite 200 Pro; Tecan, Switzerland) at 450 nm.

### Cell wall quantification assay

To assess differences in fungal cell wall composition, the same FLU-R and FLU-S *C. tropicalis* isolates selected for transcriptomic analysis were analyzed. Cell wall components—β-glucan, mannan, and chitin—were quantified using a flow cytometry-based approach as described previously (33). Briefly, both *C. tropicalis* strains were grown to logarithmic growth phase in YPD broth at 37°C. The logarithmically growing cultures were washed and stained with concanavalin A-conjugated Texas Red, Fc-hDectin-1a, and calcofluor white to quantify the mannans, glucan, and chitin, respectively. A minimum of 10,000 events were recorded for each sample, and the data were analyzed using FlowJo software (BD Biosciences). Unstained and single-stained samples served as controls, and the data were expressed as the mean fluorescence intensity (MFI) from three independent experiments.

### Macrophage and neutrophil killing assay

The ability of FLU-R strain (P1_122) to counter the phagocyte-mediated killing as compared to the FLU-S strain (P1_128) was investigated by using neutrophil and macrophage killing assay. For this, THP-1 monocytes and PLB-985 cells were cultured in RPMI-1640 medium supplemented with 10% FBS and 1% penicillin-streptomycin. THP-1 cells were differentiated into macrophage-like cells by treatment with 100 nM phorbol 12-myristate 13-acetate (PMA) for 24 h, followed by a 24-h resting period in PMA-free medium. PLB-985 cells were differentiated into neutrophil-like cells using 0.5% dimethylformamide (DMF) for 72 h. Following differentiation, PLB-985 cells were harvested and resuspended in RPMI-1640 with 1% penicillin-streptomycin (without FBS) at a concentration of 1 × 10⁷ cells/mL. Survival of *C. tropicalis* strains in THP-1 macrophages and PLB-985 neutrophils was quantified as described previously using an MOI of 1:1 (fungi to macrophages or neutrophils) (33). Survival was calculated as a percentage of viable colony-forming units (CFUs) by comparing with uninfected *C. tropicalis* strains.

### Efficacy of disinfectants against *Candida tropicalis* biofilms

We evaluated the survival of a FLU-R (P1_122) and a FLU-S (P1_128) *C. tropicalis* strain same as used in transcriptomics against six commonly used surface disinfectants. The disinfectants tested included chlorine-based sodium hypochlorite with 10% available chlorine at 0.5% and 1% (Voda Chemicals, Haryana, India), hydrogen peroxide (H₂O₂) at 0.5% (Qualigens, Maharashtra, India), 2% glutaraldehyde (Voda Chemicals, Haryana, India), 0.5% ortho-phthalaldehyde solution (CIDEX OPA, Sceptre Medical, Delhi, India), and 10% dimethyl benzyl ammonium chloride (D-125 Plus NextGen Quat, Microgen, Maharashtra, India), a quaternary ammonium compound.

To neutralize the residual activity of disinfectants, a 5% sodium thiosulfate solution was applied in an appropriate volume for 15 min. Biofilms were developed in 24-well plates (NEST Biotechnology, Jiangsu, China) following the protocol described previously (82) and incubated at 37°C for 5 days. Mature biofilms were exposed to the respective disinfectants for 10 min. Following treatment, the metabolic activity of the biofilms was quantified using the XTT reduction assay, as described above. Disinfectant efficacy was defined as a minimum of 80% reduction in XTT colorimetric readings compared to untreated control biofilms.

## Data Availability

The genome sequences of all 139 strains analyzed in the present study are accessible through BioProject number PRJNA1334599. The raw RNA-seq data of the two isolates P1_122 and P1_128 can be accessed in Gene Expression Omnibus under the accession number GSE309448.

## SUPPLEMENTAL MATERIAL

Supplemental figures mbio.00235-26-s0001.docx Fig. S1 to S8.

Table S1 mbio.00235-26-s0002.xlsx Information summary of 1,630 *Candida tropicalis* isolates used for MLST analysis in this study.

Table S2 mbio.00235-26-s0003.xlsx Details of 716 *Candida tropicalis* isolates used for whole-genome sequencing in this study.

Table S3 mbio.00235-26-s0004.xlsx Pairwise SNP comparison among 139 strains of *Candida tropicalis* and reference strain MYA-3404.

Table S4 mbio.00235-26-s0005.xlsx Nonsynonymous mutations in 33 triazole resistance-related genes in *Candida tropicalis* strains by whole-genome sequencing.

Table S5 mbio.00235-26-s0006.xlsx Detail of gene duplication in 10 genes related to antifungal resistance.

Table S6 mbio.00235-26-s0007.xlsx List of differentially expressed genes in two *Candida tropicalis* isolates exhibiting >512 mg/L fluconazole MIC differences.

Table S7 mbio.00235-26-s0008.xlsx List of significant metabolites observed in fluconazole-resistant isolate (P_122) as compared to fluconazole-susceptible isolate (P1_128).

Table S8 mbio.00235-26-s0009.docx Primers used in ERG11 sequencing and qRT-PCR.

ASM does not own the copyrights to Supplemental Material that may be linked to, or accessed through, an article. The authors have granted ASM a non-exclusive, world-wide license to publish the Supplemental Material files. Please contact the corresponding author directly for reuse.
