# Supplementary material for: Genomic insights into a clade-specific Candida tropicalis lineage with resistance to azoles and immune evasion traits
Source: mBio. 2026 Mar 11;17(4):e00235-26. doi: 10.1128/mbio.00235-26 (PMC13059750; doi:10.1128/mbio.00235-26)
Supplement: Supplemental figures — Fig. S1 to S8. [file mbio.00235-26-s0001.docx]

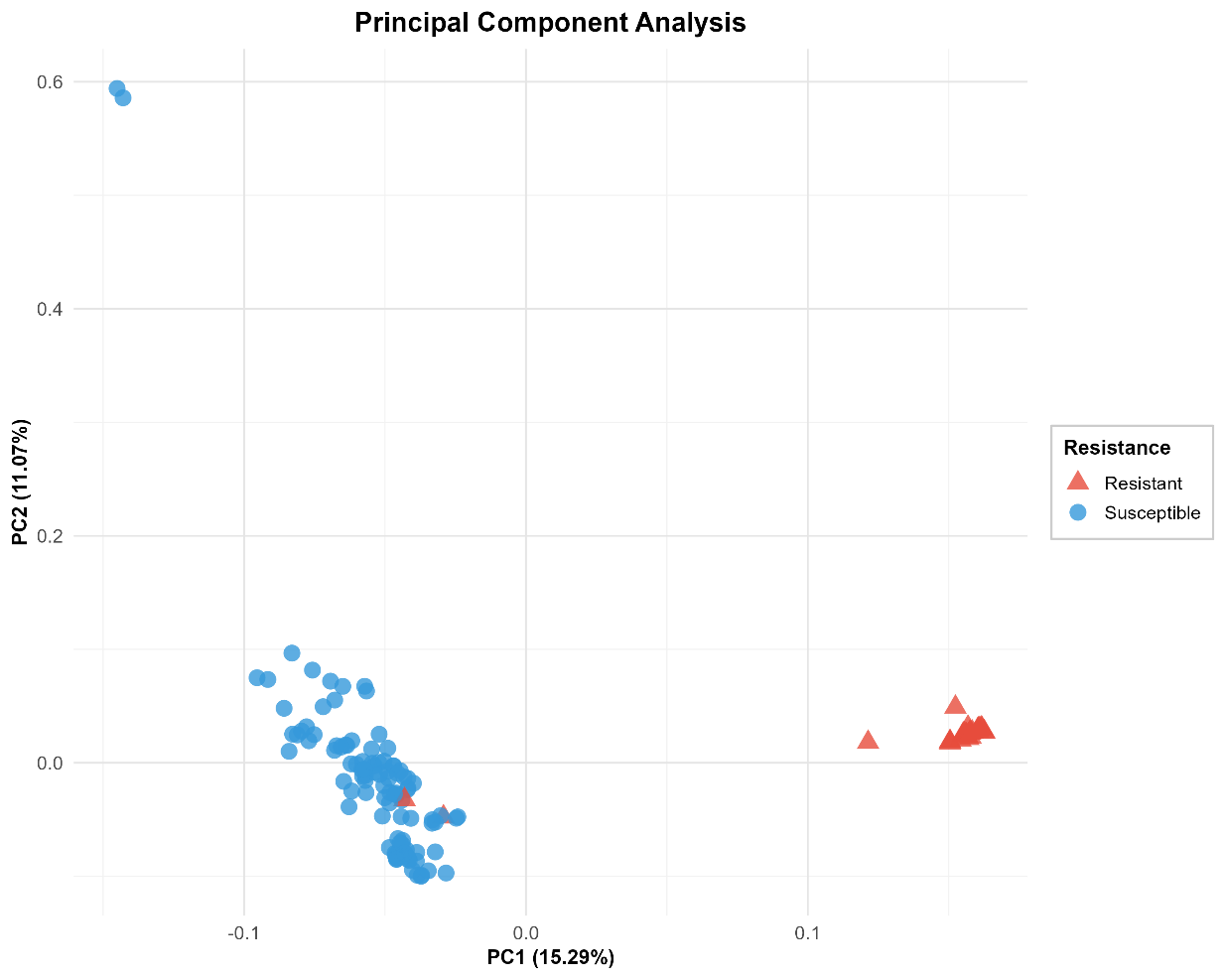


**Figure S1:** Principal component analysis based on genome-wide SNPs showing clear separation between azole-resistant (n = 30; red triangles) and azole-susceptible (n = 101; blue circles) *Candida tropicalis* isolates. The first two principal components (PC1 and PC2) explain 15.29% and 11.07% of the total genetic variance, respectively.


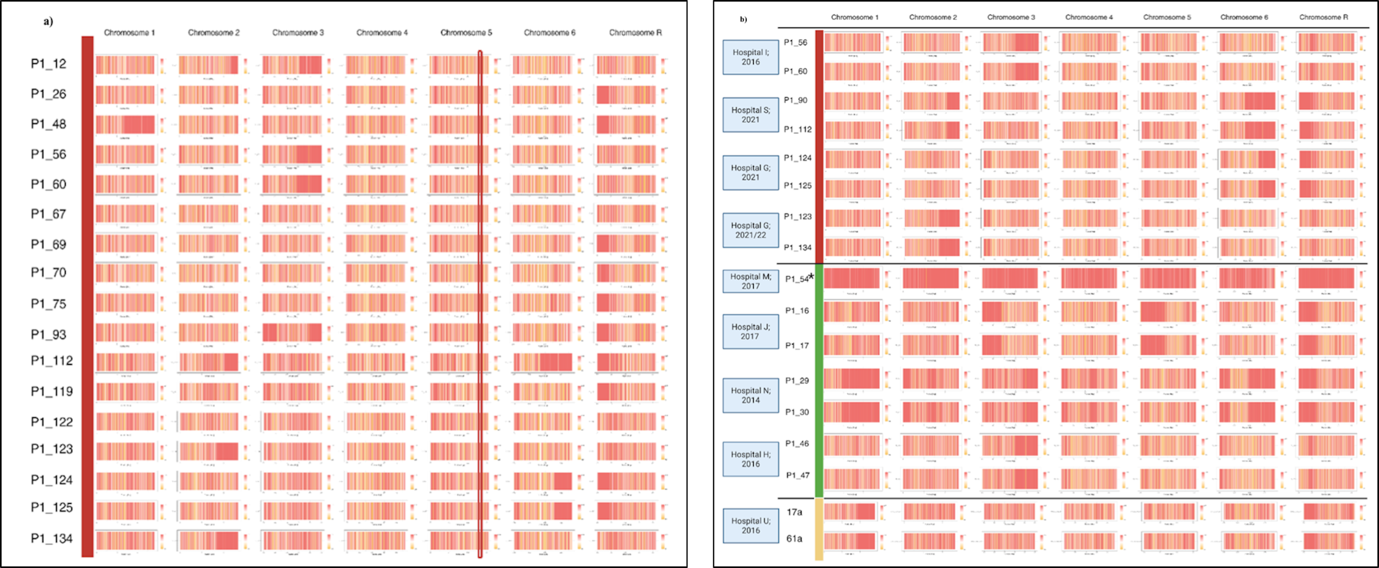


**Figure S2:** a) Scatter plots showing loss of heterozygosity (LOH) patterns in azole resistant isolates. A vertical red rectangle on chromosome 5 highlights a 900 bp LOH block (positions 1,094,487–1,095,386) encompassing the *ERG11* gene, detected in 18 fluconazole-resistant isolates. b) Scatter plots showing loss of heterozygosity (LOH) patterns in azole resistant (red panel), susceptible (green panel) and environment (yellow panel) isolates. Hospital information and year is represented in the blue box. Loss of heterozygosity blocks were inferred using the JLOH "extract" function with parameters “--min-length 100 –min-snps-per-kbp 2,6”. The asterisk mark (*) indicate a single isolate with maximum LOH at each chromosome.


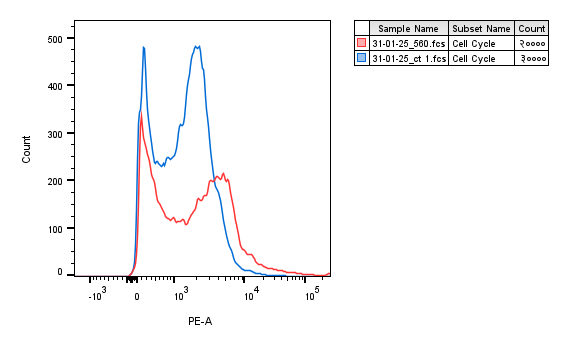


**Figure S3:** Histograms representing DNA content or cell cycle profile obtained by fluorescence-activated cell sorting. For comparison, a diploid *Candida albicans* ATCC90028 indicated by blue color was used as reference strain. *Candida tropicalis* strain had diploid genome content highlighted by red color. The x-axis represents nuclear fluorescence, and y-axis represents cell number.


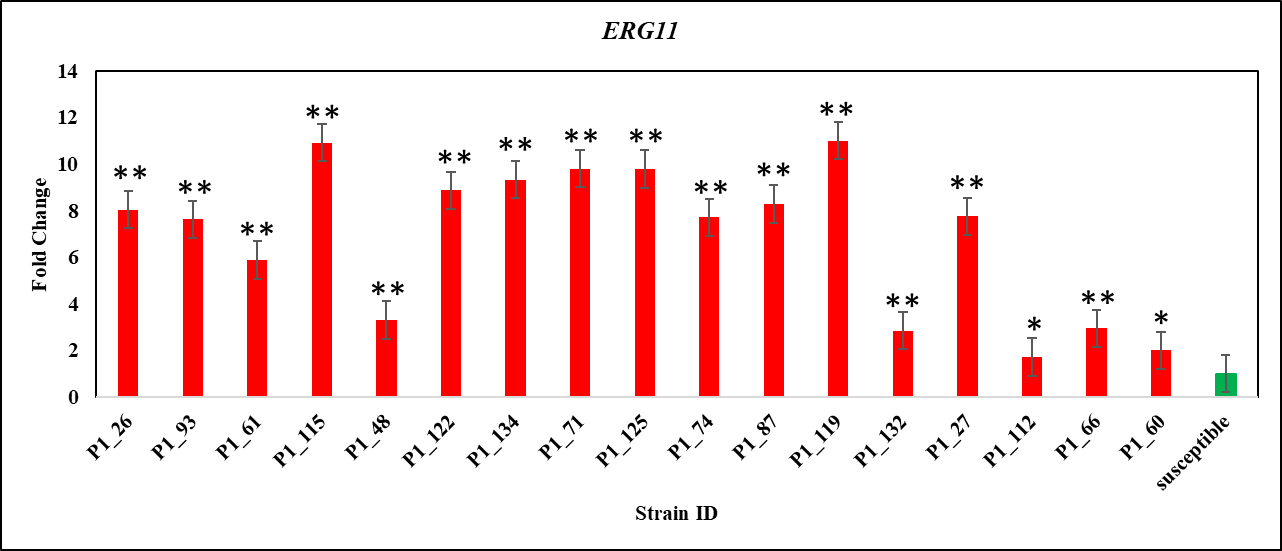


**Figure S4:** Fold change in expression of *ERG11* gene in 17 azole resistant isolates (red bars) as compared to the average expression of 13 susceptible isolates (green bar). single asterisk (*): Statistically significant (p < 0.05); double asterisk (**): Highly significant (p < 0.01).


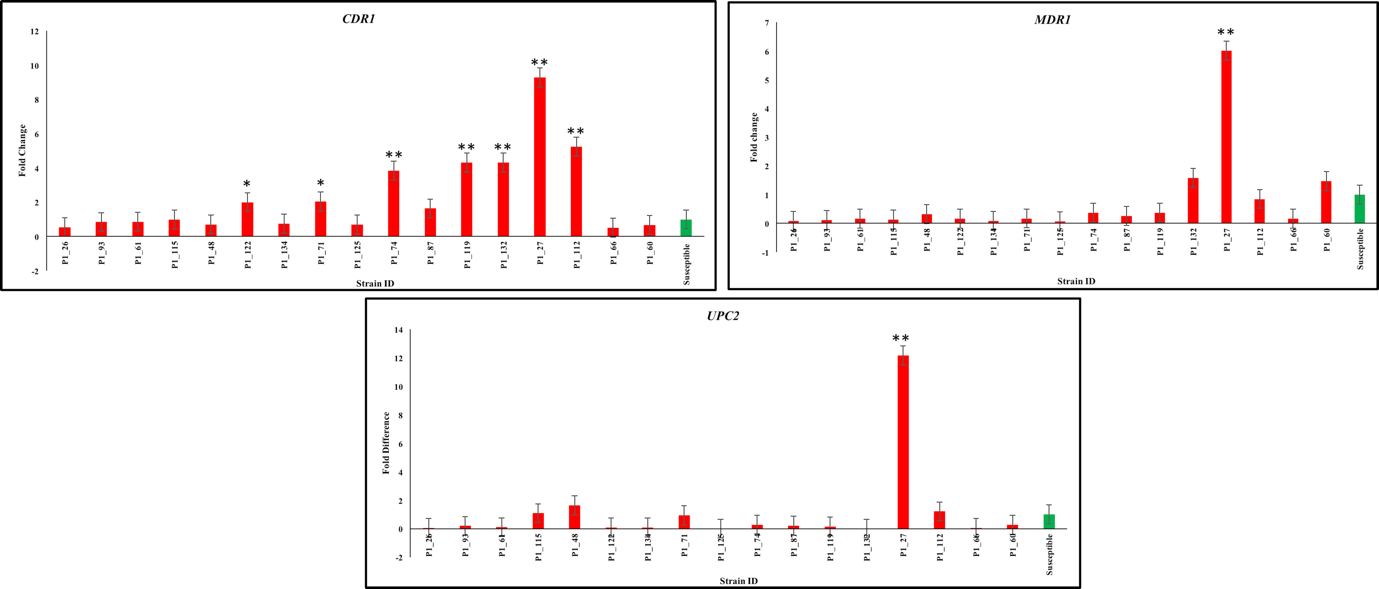


**Figure S5:** Fold change in expression of *CDR1, MDR1,* and *UPC2* genes in 17 azole resistant isolates (red bars) as compared to the average expression of 13 susceptible isolates (green bar). The single asterisk (*): Statistically significant (p < 0.05); double asterisk (**) represent highly significant (p < 0.01).


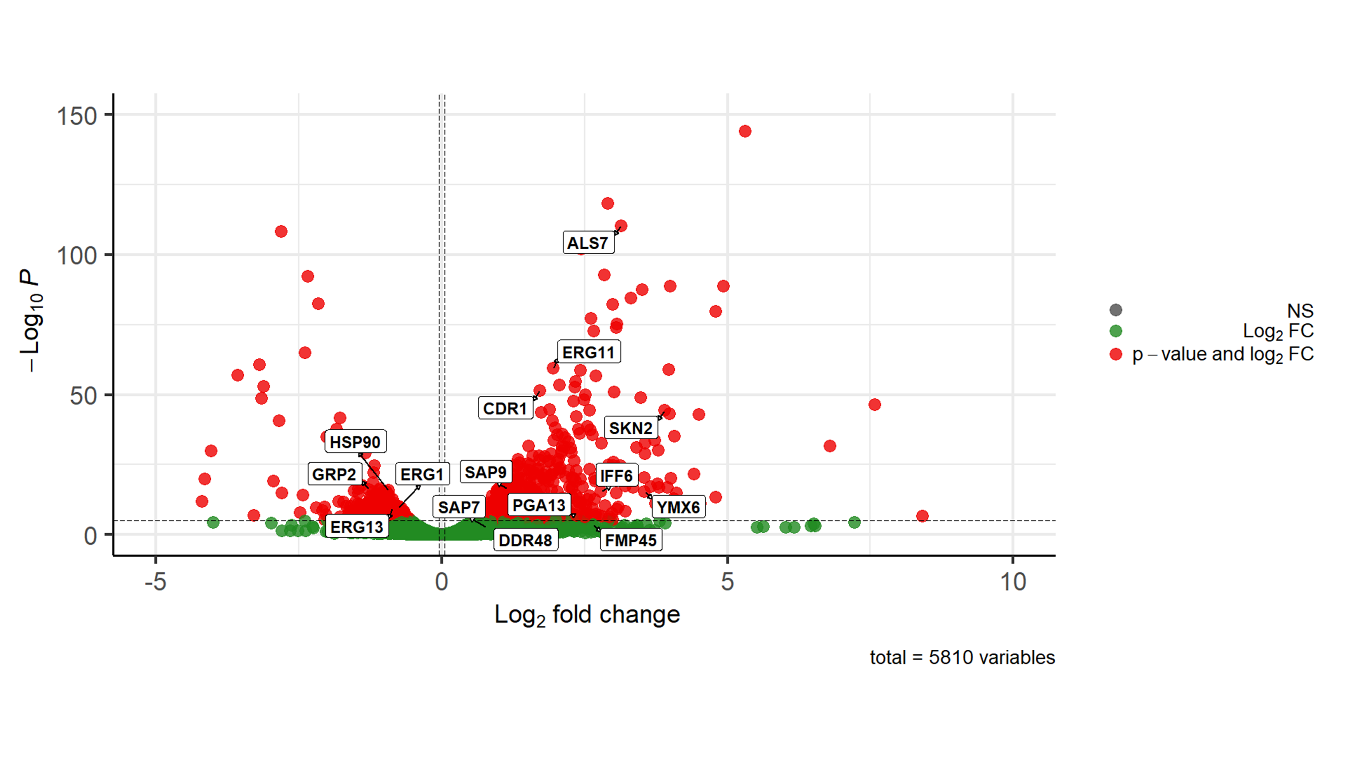


**Figure S6:** Volcano plot demonstrates the number of significantly differentially expressed genes. Both green and red dots represent significantly expressed genes at p- value ≤0.05. The labelled red dots represent genes which are expressed significantly with a fold change of ≥1.5 and involved in various biological processes.

**Figure S7**: Quantitative comparison of ergosterol content (mg/L) between FLU-R (P1_122) and FLU-S (P1_128) strains calculated using an external standard curve. Values represent mean ± SD of three biological replicates (p = 0.02).

**
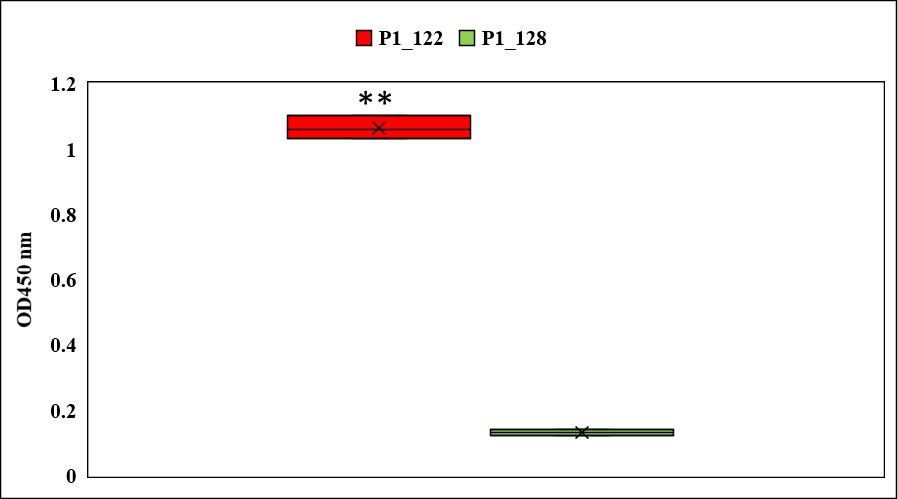
**

**Figure S8:** Box plot of 2,3-bis (2-methoxy-4-nitro-5-sulfophenyl)-5-[(phenylamino)carbonyl]-2H-tetrazolium hydroxide (XTT) values of FLU-R (P1_122) and FLU-S (P1_128) *Candida* *tropicalis* strains, the absorbance value is depicted on the y-axis.

FLU-R strains (red) exhibited significantly higher XTT values compared to FLU-S strains (green), suggesting enhanced biofilm associated metabolic activity in resistant isolates (p < 0.05). Each strain was tested in triplicate. The "X" mark inside each box indicates the mean absorbance across all replicate data in the group.
