# Supplementary material for: Genomic insights into a clade-specific Candida tropicalis lineage with resistance to azoles and immune evasion traits
Source: mBio. 2026 Mar 11;17(4):e00235-26. doi: 10.1128/mbio.00235-26 (PMC13059750; doi:10.1128/mbio.00235-26)
Supplement: Table S8 — Primers used in ERG11 sequencing and qRT-PCR. [file mbio.00235-26-s0009.docx]

**Table S8**: Primers used in *ERG11* sequencing and RT-PCR

| Primers for target gene (*ERG11*) sequencing | |
| --- | --- |
| Primer Name | Sequence (5'-3') |
| Ctrop_*ERG11*_1F | CCACTAGCAGTACCACTACC |
| Ctrop_*ERG11*_1R | GGGTATAAGCTTCTTCAGCAG |
| Ctrop_*ERG11*_2R | CCAGTAATGAGGTAATGGCAAG |
| Ctrop_*ERG11*_3R | CATAACCGGCAGAAACTAAGAC |
| Ctrop_*ERG11*_4F | CCATTGAGAGTCCCAAATACC |
| Ctrop_*ERG11*_4R | GGTACAGGTGATCTGTG |
| Primers for qRT-PCR | |
| Ctrop*_ERG 11*_F | GAGATTTGATTGATTCCTTGTTGGT |
| Ctrop*_ERG 11*_R | TGTGGTTGTTCAGCCAAATGC |
| Ctrop*_UPC2*_F | GAGTGGAACAACAACACAACAA |
| Ctrop*_UPC2*_R | TAAATCCCCTAAACCTGAAAGA |
| Ctrop*_ CDR1*_F | CCAGAGGTTTGGATTCCGCT |
| Ctrop*_ CDR1*_R | TGGCTTTGTCTGCTTTCCCA |
| Ctrop*_ MDR1*_F | GGGTGCATCATTCCAGCCTA |
| Ctrop*_ MDR1*_R | GGGATGGCAATCATCACGAG |
| Ctrop*_ ACT1*_F | TTTACGCTGGTTTCTCCTTGCC |
| Ctrop*_ ACT1*_R | GCAGCTTCCAAACCTAAATCGG |
